# Oxidative Imbalance in Psoriasis with an Emphasis on Psoriatic Arthritis: Therapeutic Antioxidant Targets

**DOI:** 10.3390/molecules29225460

**Published:** 2024-11-19

**Authors:** Rafał Bilski, Daria Kupczyk, Alina Woźniak

**Affiliations:** Department of Medical Biology and Biochemistry, Collegium Medicum in Bydgoszcz, Nicholaus Copernicus University, M. Karłowicz St. 24, PL 85-092 Bydgoszcz, Poland

**Keywords:** psoriasis, inflammation, oxidative stress, NF-κB, curcumin, antioxidants

## Abstract

Psoriasis and psoriatic arthritis (PsA) are chronic autoimmune diseases characterized by persistent inflammation and oxidative imbalance. Oxidative stress, caused by excessive production of reactive oxygen species (ROS) and dysfunction in antioxidant mechanisms, plays a critical role in the pathogenesis of both conditions, leading to increased inflammatory processes and tissue damage. This study aims to review current antioxidant-based therapeutic options and analyze oxidative stress biomarkers in the context of psoriasis and PsA. Based on available literature, key biomarkers, such as malondialdehyde (MDA), advanced glycation end-products (AGEs), and advanced oxidation protein products (AOPP), were identified as being elevated in patients with psoriasis and PsA. Conversely, antioxidant enzymes, such as superoxide dismutase (SOD), catalase (CAT), and glutathione peroxidase (GPx), showed reduced activity, correlating with symptom severity. The study also examines the efficacy of various antioxidant therapies, including curcumin, resveratrol, coenzyme Q10, and vitamins C and E, which may aid in reducing oxidative stress and alleviating inflammation. The findings indicated that antioxidants can play a significant role in alleviating symptoms and slowing the progression of psoriasis and PsA through modulation of redox mechanisms and reduction of ROS levels. Antioxidant-based therapies offer a promising direction in treating autoimmune diseases, highlighting the need for further research on their efficacy and potential clinical application.

## 1. Introduction

Psoriatic arthritis (PsA) is a chronic inflammatory disease that affects not only the skin but also the joints, leading to pain, stiffness, deformity, and numerous dermatological changes typical of psoriasis [1]. PsA affects between 6 and 42% of patients with psoriasis, making it a significant health issue for many individuals worldwide. This disease is classified as a spondyloarthropathy, characterized by chronic inflammation of the joints [2]. The etiology of PsA is multifactorial, involving both genetic and environmental factors. As a result of these factors, the immune system is activated to attack the body’s own tissues, causing chronic inflammation. The disease is more common in North America and Europe than in Asia. Females exhibit reduced radiographic progression compared to male patients; however, they experience poorer outcomes in pain, function, and exhaustion, which results in a diminished response to treatment relative to male patients. PsA typically manifests in middle-aged individuals. Nevertheless, as many as 25% of patients may experience a delayed onset. Individuals with delayed presentation are predominantly male, exhibit an extended duration of psoriasis, are obese, and possess HLA-C*06. Moreover, various studies concur that the onset of symptoms beyond 60–65 years correlates with more aggressive disease, an increased count of swollen joints, elevated acute-phase reactants, and heightened weariness. As anticipated, late onset is likewise correlated with increased comorbidity. Moreover, disparities exist regarding sex and age of onset—male patients with early-onset PsA exhibit greater axial involvement than females, whilst females demonstrate a higher prevalence of familial history of PsA [3].

The pathogenesis of psoriasis and psoriatic arthritis (PsA) remains incompletely understood; however, numerous studies indicate the key role of reactive oxygen species (ROS) and oxidative stress in its development [4,5,6,7]. ROS are molecules generated in metabolic processes within the body, such as cellular respiration, playing an essential role in intercellular signaling and immune response. However, when produced in excess, they can lead to cellular damage by oxidizing proteins, lipids, and DNA, contributing to the development of chronic inflammatory conditions [8,9].

Oxidative stress occurs when there is an imbalance between ROS production and the body’s ability to neutralize them. Antioxidant enzymes, such as superoxide dismutase (SOD), catalase (CAT), and glutathione peroxidase (GPx), play a crucial role in neutralizing excess ROS, thereby protecting cells from oxidative damage. The ROS levels are elevated in many metabolic disorders, such as diabetes, hypertension, obesity, and cardiovascular diseases, by damaging proteins and phospholipids [10]. In pathological conditions, such as psoriatic arthritis, antioxidant systems cannot effectively control ROS levels, leading to intensified inflammatory processes and tissue damage within both joints and skin [5,6].

Thus, PsA results from complex interactions between genes, the environment, and immune response mechanisms, with oxidative stress playing a crucial role. ROS stimulate the activation of immune cells, leading to increased production of pro-inflammatory cytokines, such as TNF-α, IL-6, and IL-17, which are responsible for the initiation and maintenance of the inflammatory process. Additionally, ROS affect connective tissue cells, such as fibroblasts and chondrocytes, causing dysfunction and cell death, which results in the progressive destruction of joints [11,12,13,14,15].

The significance of oxidative stress in PsA pathogenesis has been confirmed in numerous studies, showing that patients with this disease exhibit elevated levels of oxidative stress biomarkers, such as malondialdehyde (MDA), and reduced activity of antioxidant enzymes [16,17,18,19,20]. The increase in ROS levels and the reduced activity of SOD, CAT, and GPx exacerbate lipid peroxidation processes, damage proteins, and lead to DNA fragmentation, contributing to disease progression and worsening clinical symptoms.

The imbalance between ROS and antioxidant defense mechanisms is, therefore, essential for understanding the molecular mechanisms underlying PsA. Excessive ROS production not only contributes to tissue damage but can also impact other processes, such as angiogenesis and even tumor development [21], highlighting the significance of this pathway in inflammatory diseases. With the growing scientific interest in the role of oxidative stress in autoimmune and inflammatory diseases, such as PsA, more studies are focusing on potential therapeutic strategies and treatment options that could modulate this mechanism [5,22]. Treatments aimed at reducing oxidative stress, including the use of antioxidants and biological drugs that affect ROS levels and antioxidant enzyme activity, represent a promising direction in research. Such treatments could not only alleviate symptom severity but also slow disease progression, ultimately improving patients’ quality of life.

## 2. Mechanisms of Oxidative Stress in Psoriatic Arthritis (PsA)

In pathological conditions, such as PsA, excessive production of ROS significantly influences the development of inflammation. ROS can be generated in various cell types, including fibroblasts, keratinocytes, immune cells, and synovial cells, which are particularly involved in inflammatory processes within the joints [23,24,25]. One of the primary sources of ROS in cells is mitochondria, which, while generating energy in the form of ATP, also produce superoxide anions (O_2_^−^), one of the main forms of ROS [26]. Another source of ROS includes enzymes, such as NADPH oxidase, located in cell membranes, which participates in immune responses by producing peroxides, thereby increasing oxidative stress in inflamed tissues [27]. Cellular sources of reactive oxygen species (ROS) also encompass peroxisomes, which execute numerous metabolic functions: β-oxidation and α-oxidation of fatty acids, as well as the metabolism of purines and amino acids. Peroxisomes are especially abundant in the liver and kidney cells due to the involvement in detoxification and metabolic processes. In peroxisomal processes, ROS and reactive nitrogen species are produced, including O_2_, H_2_O_2_, hydroxyl radical (OH^•^), peroxynitrite (ONOO^−^), and nitric oxide (NO). The primary enzymes that facilitate the processes resulting in the production of ROS in peroxisomes are xanthine oxidoreductase and urate oxidase. Similar to mitochondria, peroxisomes possess an antioxidant defense mechanism. Consequently, ROS are primarily generated as by-products of metabolic processes, necessitating the presence of nearby systems that neutralize their oxidative effects [5].

Reactive oxygen species also play a role in activating signaling pathways responsible for chronic inflammation in PsA. Furthermore, they stimulate pathways, such as NF-κB (nuclear factor kappa-light-chain-enhancer of activated B cells) and MAPK (mitogen-activated protein kinase), which are crucial in regulating immune responses and producing pro-inflammatory cytokines [28,29,30]. NF-κB is a key transcriptional regulator controlling the expression of numerous genes involved in inflammatory responses. Its activation leads to increased production of cytokines, such as TNF-α, IL-1β, IL-6, and IL-17, which are the main mediators of inflammation in PsA [31,32,33,34]. Elevated ROS levels lead to protein, lipid, and DNA damage, resulting in cell death and tissue function impairments [35]. In joints, ROS can cause cartilage degradation, leading to joint destruction. Damage to synovial cells and fibroblasts due to ROS contributes to chronic inflammation and joint tissue degeneration, intensifying pain, stiffness, and limited joint mobility in PsA patients [7,36,37]. Additionally, ROS affect the functions of other cell types involved in inflammatory processes, such as macrophages and neutrophils [38,39]. Macrophages, which are key mediators of the inflammatory response in PsA [40], produce more pro-inflammatory cytokines and chemokines under the influence of ROS, attracting other immune cells to the inflammation site. Neutrophils, among the first cells recruited to the inflammation site, are also strongly activated by ROS, leading to further ROS production and creating an inflammatory feedback loop. It is worth emphasizing that ROS not only contribute to the initiation and maintenance of inflammation but also intensify joint destruction by activating matrix metalloproteinases (MMPs) [41,42,43]. Metalloproteinases are enzymes that degrade extracellular matrix components, such as collagen and elastin, causing cartilage and other joint structure damage. Under oxidative stress conditions, MMP activity is increased, accelerating joint degeneration and exacerbating tissue destruction. Excessive ROS production resulting from metabolic disorders and chronic inflammation initiates a cascade of reactions where oxidative stress intensifies inflammatory processes, which, in turn, lead to further ROS production. Ultimately, this pathological mechanism results in progressive joint destruction and worsened clinical outcomes for PsA patients. Therefore, modulating oxidative stress by increasing antioxidant enzyme activity or using compounds with antioxidant properties may represent a promising therapeutic strategy for treating PsA.

## 3. Dysfunction of Antioxidant Enzymes

Under physiological conditions, the body maintains a balance between ROS production and their neutralization through antioxidant mechanisms, including enzymes such as superoxide dismutase (SOD), catalase (CAT), and glutathione peroxidase (GPx) [44]. However, in inflammatory diseases, such as psoriatic arthritis (PsA), this balance is disrupted. The antioxidant enzyme system is not efficient enough to neutralize the excessive accumulation of ROS, which in turn intensifies inflammatory processes and contributes to tissue destruction.

Superoxide dismutase (SOD) serves as the first line of defense and is one of the most critical antioxidant enzymes, responsible for neutralizing the superoxide anion (O_2_^−^), one of the most reactive forms of ROS. SOD catalyzes the conversion of the superoxide anion to the less toxic hydrogen peroxide (H_2_O_2_), which is subsequently broken down by other enzymes, such as catalase and glutathione peroxidase [45]. In healthy conditions, SOD effectively protects cells from oxidative damage. Studies have shown that plasma SOD activity is significantly lower in PsA patients compared to healthy individuals. Reduced SOD activity correlates with increased ROS levels and intensified inflammatory processes in joints. Insufficient SOD activity leads to the accumulation of superoxide anions, which intensifies oxidative stress and further damages cells. The superoxide anion can react with other molecules, forming even more reactive ROS forms, such as the hydroxyl radical (OH^•^), which is particularly toxic to cells [20,46,47,48].

Catalase (CAT) is another key antioxidant enzyme that plays an essential role in neutralizing hydrogen peroxide (H_2_O_2_), produced as a result of SOD activity. Although hydrogen peroxide is relatively less reactive compared to other ROS forms, high concentrations can still lead to cellular damage. Catalase decomposes H_2_O_2_ into water and oxygen, preventing its accumulation and further oxidative damage [49]. In PsA, catalase activity is also reduced, leading to the accumulation of H_2_O_2_ in inflamed tissues. If not effectively neutralized, hydrogen peroxide can participate in Fenton reactions, leading to the formation of the hydroxyl radical, which is highly damaging to cells. Reduced catalase activity in PsA patients has been confirmed in numerous studies, which indicate that decreased enzyme activity correlates with elevated oxidative stress markers and worsened clinical symptoms [20,50,51,52].

Glutathione peroxidase (GPx) is an antioxidant enzyme that plays a crucial role in detoxifying organic peroxides and hydrogen peroxide. GPx functions in conjunction with glutathione (GSH), one of the most important low-molecular-weight antioxidants, essential for maintaining cellular redox balance. GPx catalyzes the reduction of H_2_O_2_ to water, simultaneously oxidizing glutathione. In inflammatory diseases, such as psoriasis and PsA, GPx activity is significantly reduced, impairing the cellular ability to detoxify ROS [53,54]. Decreased GPx activity increases cellular vulnerability to peroxide-induced damage, contributing to intensified inflammation and destruction of joint and skin tissues [7,55,56]. Reduced GPx activity in PsA is directly associated with symptom severity and worsened clinical outcomes in patients. Studies have demonstrated that PsA patients have significantly reduced levels of glutathione and GPx compared to healthy individuals, suggesting that antioxidant system deficiencies may play an essential role in the disease’s pathogenesis [53,57,58].

## 4. The Role of Antioxidant Enzyme Dysregulation in the Pathogenesis of Psoriatic Arthritis (PsA)

Reduced activity of antioxidant enzymes, such as SOD, CAT, and GPx, is a key factor contributing to the pathogenesis of PsA. Combined with excessive ROS production, this leads to chronic oxidative stress, which intensifies inflammatory processes and accelerates tissue destruction. High ROS levels in the joints and skin of PsA patients result in extracellular matrix degradation, DNA damage, lipid peroxidation, and impaired cellular functions [4,5,6,7].

Elevated levels of oxidative stress markers, such as malondialdehyde (MDA), and reduced activity of antioxidant enzymes are directly associated with the severity of PsA clinical symptoms, including joint deformities. Oxidative stress not only contributes to inflammation but may also lead to complications, such as cardiovascular diseases, which frequently co-occur in PsA patients [59,60,61].

## 5. Biomarkers of Oxidative Stress in PsA

Oxidative stress biomarkers play a key role in assessing the level of oxidative stress and its impact on the progression of inflammatory diseases, such as psoriatic arthritis (PsA). In PsA patients, elevated levels of reactive oxygen species (ROS) and their damaging effects on cells and tissues have been observed. These biomarkers are used both to monitor the intensity of oxidative stress and to evaluate responses to antioxidant therapies. Among the most frequently studied biomarkers are malondialdehyde (MDA), advanced glycation end-products (AGEs), and advanced oxidation protein products (AOPP) [62,63,64].

Malondialdehyde (MDA) is one of the most commonly studied biomarkers of oxidative stress, as it is a product of lipid peroxidation—a process in which ROS react with membrane lipids, leading to their damage. MDA is formed as a result of the degradation of polyunsaturated fatty acids, and its elevated concentration in plasma, synovial fluid, and urine in PsA patients indicates increased oxidative stress in these tissues [64]. Studies have shown that PsA patients have significantly higher MDA levels compared to healthy individuals, indicating heightened lipid peroxidation in joints and other inflamed tissues [16,46,57,65,66,67]. MDA is also used as a marker to evaluate therapy effectiveness. Clinical trials have observed that biological therapies, such as TNF-α and IL-17 inhibitors, significantly reduce MDA levels, indicating their positive effect on reducing oxidative stress. Monitoring MDA levels can thus serve as a therapy response indicator and help predict disease progression [68,69,70].

Advanced glycation end-products (AGEs) are another group of oxidative stress biomarkers formed by non-enzymatic reactions between sugars and proteins, lipids, or nucleic acids. This process, known as glycation, produces stable compounds that accumulate in tissues, exacerbating inflammation. AGEs are strongly associated with chronic inflammatory diseases, including PsA, as their accumulation in inflamed tissues leads to cell damage and intensification of inflammatory responses [71]. AGEs can bind to receptors on cell surfaces, such as the receptor for advanced glycation end-products (RAGE), activating inflammatory pathways, including NF-κB, and increasing pro-inflammatory cytokine production. This process contributes to chronic inflammation and tissue damage in PsA. Studies have shown that PsA patients have higher AGE levels compared to healthy individuals, correlating with increased clinical symptoms and disease progression [71,72]. AGEs are also significant in terms of vascular complications, often coexisting in PsA patients. Accumulation of AGEs in blood vessel walls contributes to atherosclerosis and other cardiovascular diseases, which are common complications in inflammatory diseases, including PsA. Therefore, monitoring AGE levels may not only assess inflammation but also help predict cardiovascular risk in PsA patients [71].

Advanced oxidation protein products (AOPP) are another group of biomarkers that play a crucial role in monitoring oxidative stress in PsA patients. AOPP are formed by oxidative modifications of proteins, mainly albumin, which are essential in the response to excessive ROS production. This process leads to changes in protein structure and function, contributing to the intensification of inflammatory and degenerative processes [63,64]. Studies have shown that AOPP levels are significantly elevated in PsA patients, suggesting that oxidative stress contributes to protein damage in affected joints and other tissues. Increased AOPP levels are also correlated with the severity of PsA clinical symptoms and progressive joint destruction. Additionally, AOPP can act as mediators of inflammatory responses, stimulating cytokine production and attracting immune cells to the inflammation site, which further intensifies the inflammatory process [16,63,64,73]. Similar to MDA and AGEs, AOPP levels can be monitored to assess therapy effectiveness. A decrease in AOPP levels in response to treatment indicates a reduction in oxidative stress and an improvement in the clinical condition of PsA patients. Monitoring AOPP levels is particularly important in assessing the long-term impact of therapy on disease progression and its complications [6].

Oxidative stress biomarkers, such as MDA, AGEs, and AOPP, play a crucial role in monitoring PsA progression and therapy effectiveness. Their elevated levels indicate heightened oxidative stress, leading to tissue destruction and inflammation intensification. Monitoring these biomarkers can provide valuable insights into a patient’s health status, disease severity, and treatment response. Moreover, these biomarkers can be used as prognostic tools, allowing for the prediction of complications, such as cardiovascular disease, which is a common issue in inflammatory diseases, including PsA. Therefore, in addition to assessing joint inflammation, monitoring oxidative stress biomarkers offers a broader view of overall patient health and aids in the development of more personalized therapeutic strategies. A summary of research findings in this area is provided in Table 1.

## 6. The Impact of Therapy on Oxidative Stress

Oxidative stress plays a critical role in the pathogenesis of psoriatic arthritis (PsA), making modulation of this process a potentially significant component of disease treatment. Recently, therapies aimed at reducing reactive oxygen species (ROS) production and enhancing the activity of antioxidant enzymes, such as superoxide dismutase (SOD), catalase (CAT), and glutathione peroxidase (GPx), have gained increasing interest. Among the available treatments for PsA are both biological therapies and antioxidant supplementation, which may support oxidative stress reduction and help prevent tissue damage [86,87,88].

Biological therapies used in PsA treatment play a vital role in modulating oxidative stress by regulating immune responses and reducing ROS production. Tumor necrosis factor-alpha (TNF-α) inhibitors, such as etanercept, adalimumab, and infliximab, as well as interleukin-17 (IL-17) inhibitors, such as secukinumab, significantly reduce inflammation by blocking pro-inflammatory cytokines. These therapies not only mitigate inflammation but also modulate the activity of antioxidant enzymes, leading to a decrease in ROS levels and an improvement in redox balance in affected tissues [89,90,91,92,93,94,95]. TNF-α inhibitors, such as etanercept, reduce oxidative stress by lowering the activity of pro-inflammatory cytokines that stimulate ROS production. TNF-α is one of the key cytokines activating signaling pathways that lead to ROS production, and its inhibition lessens the intensity of the inflammatory response and the accumulation of ROS in tissues. Studies have shown that PsA patients treated with TNF-α inhibitors exhibit significantly reduced ROS levels and oxidative stress biomarkers, such as MDA. These therapies also enhance the activity of SOD, CAT, and GPx, further reducing oxidative stress and protecting tissues from additional damage [93,96]. Secukinumab, an IL-17 inhibitor, also plays an essential role in modulating oxidative stress in PsA patients. IL-17 is a cytokine pivotal to the pathogenesis of various autoimmune diseases, including PsA. Its inhibition by secukinumab leads to a reduction in the inflammatory response and a decrease in ROS production by immune cells. Research has shown that treatment with secukinumab reduces ROS levels and increases SOD activity, translating into improved clinical outcomes for patients [97].

In addition to biological therapies, antioxidant therapies have gained significant interest as potential adjunct treatments for PsA by directly reducing ROS levels and enhancing the activity of antioxidant enzymes. Antioxidants, such as vitamins C and E, coenzyme Q10, glutathione, and polyphenols, are used to neutralize ROS and prevent oxidative damage [86,98,99]. Vitamin C (ascorbic acid) is a potent antioxidant that acts as an electron donor, neutralizing ROS, including hydrogen peroxide (H_2_O_2_) and hydroxyl radicals (OH·). This vitamin plays a crucial role in protecting cells from oxidative stress by scavenging superoxide and hydrogen peroxide and regenerating other antioxidants, such as vitamin E. It also acts as a cofactor of α-ketoglutarate-dependent nonheme iron dioxygenases in collagen synthesis [100]. Clinical studies have shown that vitamin C supplementation in patients with psoriasis reduces oxidative stress biomarkers, such as malondialdehyde (MDA), and increases the activity of antioxidant enzymes, including glutathione (GSH), SOD, and CAT. Vitamin C may also modulate the immune response, contributing to reduced inflammation [101,102,103]. Vitamin E (α-tocopherol) is another powerful antioxidant that plays a key role in protecting cell membranes from lipid peroxidation. Vitamin E, an effective peroxyl radical scavenger, serves as a chain-breaking antioxidant that inhibits the propagation of free radicals in membranes and plasma lipoproteins. Peroxyl radicals (ROO^•^) react with vitamin E (Vit E-OH) at a rate 1000 times faster than with polyunsaturated fatty acids (PUFA) upon formation. The hydroxyl group of tocopherol interacts with the peroxyl radical, resulting in the formation of lipid hydroperoxide and the tocopheryl radical (Vit E-O). The tocopheryl radical (Vit E-O) interacts with vitamin C (or other hydrogen donors, AH), oxidizing the latter and restoring vitamin E to its reduced form. Studies on PsA have demonstrated that vitamin E supplementation leads to reduced ROS levels and clinical improvement in patients. Vitamin E works synergistically with vitamin C, enhancing their combined antioxidant effects [100,103,104]. Coenzyme Q10 is another potent antioxidant that acts at the mitochondrial level, where it neutralizes ROS generated during energy production. Coenzyme Q10 also regenerates other antioxidants, such as vitamin E, which increases its protective potential. Studies have shown that coenzyme Q10 supplementation reduces MDA levels and improves antioxidant enzyme function, leading to symptom reduction in PsA [103,104,105,106].

Increasing research focuses on developing novel targeted therapies that can modulate oxidative stress through direct intervention in cellular redox mechanisms. Next-generation biologics, such as inhibitors of NF-κB and MAPK signaling pathways—activated by ROS—offer a promising new therapeutic strategy for PsA treatment. Blocking these pathways can reduce ROS production and lower pro-inflammatory cytokine levels, thereby decreasing oxidative stress and halting inflammatory processes [107,108,109,110].

Additionally, there is growing interest in the use of natural substances with strong antioxidant properties, such as curcumin, resveratrol, and green tea catechins. These compounds exhibit potent antioxidant and anti-inflammatory effects, making them valuable for supporting the treatment of inflammatory diseases, including PsA. Preliminary studies on the use of curcumin have shown that it can reduce levels of ROS and oxidative stress biomarkers, leading to a reduction in the severity of clinical symptoms. Curcumin belongs to the group of diarylheptanoids. This class of chemicals has demonstrated anti-inflammatory, antioxidant, anticancer, antiviral, hepatoprotective, and neuroprotective properties. These chemicals limit lipopolysaccharide (LPS)-stimulated cellular responses in macrophages, obstruct reactive oxygen species and nitrite generation induced by LPS, and suppress inflammatory cytokines IL-1β and IL-6 without causing hazardous side effects [111,112,113,114,115,116]. Moreover, Kan-Lu-Hsiao-Tu-Tan (KLHTT) extract, derived from traditional Chinese medicine, has demonstrated antioxidant and anti-inflammatory effects in a collagen-induced mouse arthritis model. KLHTT reduced levels of reactive oxygen species (ROS), including superoxide anions and hydrogen peroxide, which play a crucial role in PsA pathogenesis. Additionally, it lowered pro-inflammatory cytokines, such as IL-1β, IL-6, IL-17A, and TNF-α. The extract also reduced the numbers of Th1 and Th17 cells and the levels of anti-collagen antibodies, leading to an improvement in clinical symptoms of arthritis in mice [117,118]. The study conducted by Ramanan et al. proved that 3-aryl iso-coumarins are potent inhibitors for 5-lipoxygenase in vitro and prostaglandin E_2_ in vivo. This research, if extended in the future, could contribute to the problem of psoriasis and other autoimmune diseases [119]. A summary of new therapeutic approaches is presented in Table 2.

Modifying dietary habits can enhance the quality of life for patients by alleviating skin lesions and diminishing the risk of other disorders. A low-calorie diet is advised for those with excessive body weight. Individuals afflicted with psoriasis should restrict their consumption of saturated fatty acids and substitute them with polyunsaturated fatty acids from the omega-3 family, known for their anti-inflammatory properties. The incorporation of antioxidants, including vitamins A, C, E, carotenoids, flavonoids, and selenium, is crucial in dietary therapy for individuals with psoriasis. Supplementation of vitamin D is also advised. Certain scientists propose that alternate diets positively influence the progression of psoriasis. The diets encompass a gluten-free diet, a vegetarian diet, and a Mediterranean diet. Dietary therapy for individuals with psoriasis should be customized to complement pharmaceutical treatment. For example, folic acid supplementation is administered to those undergoing methotrexate therapy [135].

The modulation of oxidative stress through biological therapies, antioxidant treatments, and novel therapeutic approaches plays a crucial role in managing PsA. Reducing ROS levels and increasing antioxidant enzyme activity can lead to a decrease in disease severity, improved joint function, and inhibition of progressive tissue destruction. Combining biological therapies with antioxidant supplementation represents a promising therapeutic strategy that not only alleviates symptoms but also slows disease progression and prevents complications. Further research on the impact of these therapies on oxidative stress in PsA may provide new therapeutic tools that are more effective in controlling the disease and improving patients’ quality of life.

## 7. Limitations

This review has several limitations that should be acknowledged. Firstly, the studies included primarily relied on cross-sectional data, which limited the ability to infer causative relationships between oxidative stress markers and the progression of psoriasis and psoriatic arthritis (PsA). Most available research data were observational, which restricted a direct understanding of how antioxidant therapies might influence disease course over time. Furthermore, there was substantial heterogeneity in the methodologies of the included studies and variability in oxidative stress biomarker levels among patients, which challenged the comparability of findings across studies. This variability complicated drawing definitive conclusions about the relative efficacy of antioxidant treatments and the specific roles of individual oxidative markers in disease mechanisms. Finally, while this review highlighted potential antioxidant therapies, the long-term safety and efficacy of such treatments in psoriasis and PsA remain unclear due to the limited number of longitudinal studies. Further research, including well-designed, randomized controlled trials, is necessary to confirm the therapeutic benefits of antioxidants and clarify their role in the management of oxidative stress in psoriasis and PsA.

## 8. Conclusions

Understanding the molecular mechanisms associated with ROS overproduction and its impact on the development of autoimmune diseases, such as PsA, is essential for designing effective therapies that could not only alleviate symptoms but also slow disease progression. Assessing oxidative stress biomarkers may be crucial in evaluating treatment efficacy and monitoring disease progression. Furthermore, it can provide valuable insights not only into joint inflammation but also into the overall cardiovascular risk in PsA patients. The application of biological and antioxidant therapies may have a beneficial effect on reducing oxidative stress and protecting tissues from further damage. Future research should, therefore, focus on gaining a deeper understanding of the mechanisms underlying redox imbalance in PsA and developing new therapeutic strategies that can effectively modulate oxidative stress. Additionally, further studies on novel therapeutic approaches should focus on ROS-activated signaling pathways, which could enhance the effectiveness of these treatments. Monitoring oxidative stress biomarkers and assessing redox balance could become a key component in developing personalized therapies that more effectively control disease symptoms and improve patient health.

## Figures and Tables

**Table 1:** Summary of oxidative stress markers and the state of the natural antioxidant barrier in patients with psoriasis and PsA.

Antioxidant/Biomarker	Result in Patients	References
MDA	Increased	[16,19,52,66,74,75,76]
	No differences	[17,18]
SOD	Decreased	[20,47,52,76]
	Increased	[77]
CAT	Decreased	[50,51,75,78]
GPx	Decreased	[77]
Uric acid	Decreased	[79]
AGE	Increased	[16,80]
AOPP	Increased	[16,73,81]
	No differences	[17]
TAS (total antioxidant status)	Decreased	[75,82]
TAC (total antioxidant capacity)	Decreased	[76,83]
LOOH (lipid hydroperoxides)	Increased	[16,73,84]
Total oxidant status (TOS)	Increased	[16,18,75,82,85]
Oxidative Stress Index (OSI)	Increased	[16,82]

**Table 2:** New therapeutic targets in psoriasis and PsA oxidative stress treatment.

Therapeutic Agent	Mechanism of Action	Therapeutic Effect	References
Curcumin	Inhibits NF-κB and MAPK pathways, reducing the production of pro-inflammatory cytokines (IL-6 and TNF-α).	Reduction of inflammation, decrease in oxidative stress, and improvement in psoriasis and PsA symptoms.	[88,111,120,121,122]
Coenzyme Q10, vitamin E, selenium	Increases activity of SOD and CAT and decreases oxidative stress.	Improvement of redox balance and accelerated clinical recovery.	[104,106,123,124,125,126]
Resveratrol	Neutralizes ROS, inhibits NF-κB pathways, stimulates autophagy, and suppresses macrophage infiltration.	Reduction of oxidative stress and enhancement of immune response.	[28,127,128,129]
Quercetin	Decreases ROS production and inhibits NF-κB activity.	Reduction of oxidative stress and improvement of keratinocyte function.	[130,131,132,133]
Vitamin C	Neutralizes H_2_O_2_ and hydroxyl radicals (OH·).	Improvement of antioxidant enzyme function and reduction of lipid peroxidation.	[125,134,135]
Green tea extract (catechins)	Neutralization of ROS and stimulation of antioxidant enzyme activity.	Reduction of inflammation and oxidative stress.	[126,136]
Astilbin	Increases Nrf2 accumulation, activates antioxidant-related proteins, and reduces IL-17-dependent T lymphocytes’ accumulation.	Reduction of oxidative stress and improvement in clinical outcomes.	[137,138,139]
Bilirubin	High biocompatibility, skin penetration, and neutralizes ROS.	Reduction of ROS accumulation and improvement of skin condition in psoriasis.	[87,140,141,142]
Aloe vera	Inhibits NF-κB, MAPK, and PI3K signaling pathways, along with reducing iNOS, IL-6, and IL-1β synthesis in macrophages, or lowering prostaglandin E2 levels through COX inhibition.	Reduction of oxidative stress and enhancement of skin lesion healing.	[143,144,145]
Ozone therapy	Inhibits the NF-κB pathway.	Reduction of symptoms in skin lesions and joint inflammation.	[146,147]

## Data Availability

No new data were created or analyzed in this study.
